# Associations between socioeconomic status and primary total knee joint replacements performed for osteoarthritis across Australia 2003–10: data from the Australian Orthopaedic Association National Joint Replacement Registry

**DOI:** 10.1186/1471-2474-15-356

**Published:** 2014-10-28

**Authors:** Sharon L Brennan, Stephen E Lane, Michelle Lorimer, Rachelle Buchbinder, Anita E Wluka, Richard S Page, Richard H Osborne, Julie A Pasco, Kerrie M Sanders, Kara Cashman, Peter R Ebeling, Stephen E Graves

**Affiliations:** School of Medicine, Deakin University, Geelong, Australia; NorthWest Academic Centre, Department of Medicine, The University of Melbourne, Melbourne, Australia; Barwon Health Biostatistics Unit, Barwon Health, The Geelong Hospital, Geelong, Australia; Data Management and Analysis Centre, Australian Orthopaedic Association Joint Replacement Registry, Adelaide, Australia; Monash Department of Clinical Epidemiology, Cabrini Hospital, Melbourne, Australia; Department of Epidemiology and Preventive Medicine, Monash University, Melbourne, Australia; Barwon Orthopaedic Research Unit, Barwon Health, Geelong, Australia; Public Health Innovation, Deakin Population Health Strategic Research Centre, Deakin University, Melbourne, Australia; Department of Medicine, Western Health, Melbourne, Australia; Australian Institute for Musculoskeletal Science, The University of Melbourne, Melbourne, Australia; Australian Orthopaedic Association Joint Replacement Registry, Adelaide, Australia; Epi-Centre for Healthy Aging, Deakin University, C/-Barwon Health, Ryrie Street, Geelong, VIC 3220 Australia

**Keywords:** Arthroplasty, Socioeconomic status, Knee joint

## Abstract

**Background:**

Relatively little is known about the social distribution of total knee joint replacement (TKR) uptake in Australia. We examine associations between socioeconomic status (SES) and TKR performed for diagnosed osteoarthritis 2003–10 for all Australian males and females aged ≥30 yr.

**Methods:**

Data of primary TKR (n = 213,018, 57.4% female) were ascertained from a comprehensive national joint replacement registry. Residential addresses were matched to Australian Census data to identify area-level social disadvantage, and categorised into deciles. Estimated TKR rates were calculated. Poisson regression was used to model the relative risk (RR) of age-adjusted TKR per 1,000py, stratified by sex and SES.

**Results:**

A negative relationship was observed between TKR rates and SES deciles. Females had a greater rate of TKR than males. Surgery utilisation was greatest for all adults aged 70-79 yr. In that age group differences in estimated TKR per 1,000py between deciles were greater for 2010 than 2003 (females: 2010 RR 4.32 and 2003 RR 3.67; males: 2010 RR 2.04 and 2003 RR 1.78).

**Conclusions:**

Identifying factors associated with TKR utilisation and SES may enhance resource planning and promote surgery utilisation for end-stage osteoarthritis.

**Electronic supplementary material:**

The online version of this article (doi:10.1186/1471-2474-15-356) contains supplementary material, which is available to authorized users.

## Background

It is well-documented that for most causes of mortality and morbidity, a socioeconomic gradient exists [1–4]; arthritis appears to be no exception [5–8]. In Australia, national data from 2004–5 showed an inverse association between the prevalence of self-reported doctor diagnosed osteoarthritis (OA) and socioeconomic status (SES). The OA prevalence in quintiles 1 through 5 (where quintile 1 is the most disadvantaged) was 7.3%, 7.0%, 6.4%, 6.2% and 5.7%, respectively [9]. Similar associations between arthritis overall, and rheumatoid arthritis, and SES were observed Australia-wide in 2007–8 [10]. We have previously reported using multi-level analysis that a 42% increased likelihood of arthritis existed for those of greater social disadvantage compared to those who are more advantaged, even after accounting for disadvantage measured at the individual- and household-levels [5]. Furthermore, these differences were independent of advancing age and female sex; both factors that are associated with an increased prevalence of OA.

The inverse association between SES and arthritis prevalence is also observed when examining the surgical intervention for severe end-stage OA of total joint replacement; a common, cost-effective elective procedure shown to relieve pain and improve quality of life [11, 12]. Variations in the utilization of joint replacement procedures have been reported across SES in high income countries such as England [7, 13–16], United States of America [17], Italy [18] and across different geographic regions [19–23]. However, in comparison to other countries, there are few data examining SES and utilization of joint replacement over time in Australia. An examination of hospital separations over a 12-month period to identify knee joint replacements (n = 27,872) by Dixon et al. [23] showed that people residing in the most disadvantaged areas of Australia had more knee replacements than those in the most advantaged areas. Dixon et al. had earlier reported this same pattern between knee replacements and SES in England over a 10-year period [13].

Given the greater prevalence of OA for lower SES, it is plausible to expect an increased need for joint replacement in those population groups. However, it is equally plausible that socially disadvantaged individuals may be less likely to utilise joint replacement surgery for other reasons, two of which may be patient preferences [24, 25], and/or the significant indirect costs documented to be associated with recovery post-surgery. Socially disadvantaged individuals may have limited social support, and less accumulated wealth on which to draw for home modifications and temporary personal or home care needs associated with post-surgery dependence. Furthermore, in the Australian health care system where a mix of public and private sector providers deliver health services, there are more likely to be longer waiting lists in the public sector as opposed to limited wait time for individuals with private healthcare cover. Ultimately, socially disadvantaged individuals who rely upon the public sector for healthcare may be the same group that are least able to rebound strongly from the setbacks associated with time recuperating and/or reduced access to financial resources during the recovery time. Thus, despite having access to surgery through the public healthcare system, socially disadvantaged individuals may be less likely to utilise this option for end-stage disease compared to more advantaged individuals.

Taken in context, it is important to understand the socioeconomic patterning of joint replacement utilisation over the years, in order to project need and identify whether there may be improving or worsening health equity with regards to the utilisation of surgery by those of greatest need [13]. Given that no temporal patterns of joint replacements in different socioeconomic groups are known for the Australian population, nor whether any disparities exist in surgery uptake, we present the first data to examine the association between SES and the utilization of primary total knee replacement (TKR) performed for OA in adults for 2003–10 using data from a comprehensive national joint replacement registry, whilst taking into account advancing age and sex differences in OA prevalence.

## Methods

### Australian Orthopaedic Association National Joint Replacement Registry

The Australian Orthopaedic Association National Joint Replacement Registry (AOA NJRR) commenced in September 1999, funded by the Commonwealth Government through the Department of Health and Ageing, and was introduced in a state-by-state approach that was completed nationally in 2002 [26]. The AOA NJRR monitors the performance and outcome of hip and knee replacement surgery Australia-wide and receives voluntary cooperation from all hospitals undertaking joint replacement surgeries performed within both the public and private health systems. Data are matched and verified by cross-linking registry data with government separation data for all arthroplasty procedures. This verification process has established that the Registry receives information on more than 99% of all joint replacement operations. The database has been validated against health department unit record data using a sequential multi-level matching process and coupled with the retrieval of unreported procedures, the AOA NJRR is the most complete and extensive set of joint replacement data in Australia [26].

For these analyses, incident primary TKR was defined as primary replacement of the tibiofemoral joint surfaces and in some cases also the patellofemoral joint. Primary partial knee joint replacement and revision surgeries were excluded from these current analyses, due to different reasons for utilisation.

### Socioeconomic status

To determine area-based SES, we matched the full residential address of each patient to the corresponding Australian Bureau of Statistics (ABS) Census Collection District (CCD); areas that incorporate approximately 250 households. ABS reference data were used to determine the Socio-Economic Indexes For Areas (SEIFA) value from the 2001 census for each joint replacement made during 2003–5, or the 2006 census for each joint replacement made during 2006–10. We applied the Index of Relative Socioeconomic Advantage and Disadvantage (IRSAD) for this analysis, in which decile 1 represented the most disadvantaged and decile 10 represented the most advantaged. Validation of the SEIFA index was undertaken by analysts from the ABS Regional Offices and also an external peer review of the variables and methodology used in SEIFA 2006 was performed by a group of academic and policy research experts who were skilled in socioeconomic modelling and analysis [27]. Variables included in the SEIFA were validated by summing SEIFA variables at the small area to the State totals, which were then compared to published or independently created figures [27]. The ABS indicates principal components analysis, the technique applied to develop and weight the scores, has shown to be reliable [27, 28]. In 2001 and 2006, approximately 4% (n = 1,514) and 3% (n = 1,256), respectively, of CCDs could not be given a SEIFA score for reasons which included: no usual residents (which accounted for 49% of excluded CCDs), CCDs where >80% of people lived in non-private dwellings, fewer than 10 people residing in an area, fewer than five employed people in an area, five or fewer occupied private dwellings in an area, or areas in which non-response to Census questions including occupation, labour force status, type of educational institution attending, or non-school qualifications exceeded 70% [29, 30]. In the AOA NJRR patient dataset, SEIFA values were unavailable for 6% of the patients, and were thus excluded. Reasons for these missing data are unknown but could be due to the majority of joint replacement patients being (i) older and (ii) having an increased propensity to reside in non-private dwellings such as retirement villages/nursing homes. Without a residential address we were unable to cross-reference with ABS data to ascertain a valid measure of SES. TKR was performed on only a small number of patients aged 10–29 years (57.0% female) and so these were also excluded from the analysis. The AOA NJRR Data Review Committee approved the study.

### Statistical analysis

The residential address of each patient undergoing a joint replacement surgery performed during 2003–5 was matched to the 2001 census and during 2006–10 was matched to the 2006 census. The population at risk in each 10-year age group and SES decile for men and women were calculated using ABS population data, with the assumption made that these proportions were consistent across the study period for all SES deciles. Using the growth of the total Australian population each year, and assuming that population growth within SES deciles occurs at the same rate we calculated the total population in each SES decile. To calculate the population at risk, we combined the estimates of total population in SES deciles each year, along with the age by sex by year population proportions, to give the estimates of the population at risk of joint replacement in each year according to age, sex and SES.

In order to account for the possibility that proportions may vary across years, and the increased OA prevalence observed in older age groups, and also in women compared to men, Poisson regression was used to model the relative risk of primary TKR per unit time stratified by sex across SES deciles, adjusting for age group (as a categorical variable) and year of procedure. Primary interest was in the effects of sex and SES decile (and the interaction between the two) on primary TKR, whilst modelling for age and year of procedure; this was used as the initial model, with further higher order interaction terms chosen through a stepwise approach to improve model fit. The best model fit based on AIC was:

$$\begin{array}{l} \log\left(N\right)=\log\left(PAR\right)+\mathrm{int}\text{ercept}+\text{age}\;\text{group}+\text{sex}\\ \;+SES\;\text{decile}+\text{year}\;\text{of}\;\text{procedure}\\ \;+SES\;\text{decile}\times \text{sex}+\text{age}\;\text{group}\\ \;\times (\text{year}\;\text{of}\;\text{procedure}+SES\;\text{decile}\\ \;+\text{sex})+\text{year}\;\text{of}\;\text{procedure}\\ \;\times SES\;\text{decile}+\text{error} \end{array}$$

where N is the number of observed primary TKR and PAR the population at risk.

Despite there being no (statistically significant) interaction between sex and year of procedure, given the difference in OA prevalence between sexes it was important for ease of interpretation to examine whether the rates of TKR varied within sex across different SES deciles, therefore post-hoc estimates of relative rates were calculated, along with 95% confidence intervals. Goodness of fit, and model assumptions, were tested using the Residual Quantile-Quantile Plot to assess normality. Analyses were performed using R version 2.15.0 (R Foundation for Statistical Computing, Vienna, Austria) [31].

## Consent

The AOA NJRR is funded by the Commonwealth Government through the Department of Health and Ageing, and receives voluntary cooperation from all Australian hospitals undertaking joint replacement surgeries performed within both the public and private health systems. The AOA NJRR Data Review Committee, as a Federal Quality assurance Activity under the Health Act of 1973, approved this study, the waiver of patient consent for use of these data, and the publication of this report.

## Results

During the years 2003–10, 213,018 TKR surgeries were performed (57.4% female). Table 1 presents the total numbers and rate of primary TKR by SES deciles, with sexes combined, age-standardised to the 2006 population at risk in each SES decile. For both males and females, surgery utilisation was greatest in the 70–79 year age group. A negative relationship was observed between the proportions of TKR and SES, which was consistent for both sexes in the age groups of 50–59, 60–69 and 70–79 years.

**Table 1:** **Total primary TKR numbers for males and females combined (57.4% female) across deciles of socioeconomic status (SES), as measured by the Index of Relative Socioeconomic Advantage and Disadvantage (IRSAD), for Australia 2003–10**

Age (yr)	Deciles of SES									
	1*	2	3	4	5	6	7	8	9	10
30-39	29 (0.02)	36 (0.03)	28 (0.02)	23 (0.02)	35 (0.02)	25 (0.02)	34 (0.02)	26 (0.02)	21 (0.01)	10 (0.01)
40-49	466 (0.34)	481 (0.34)	403 (0.28)	422 (0.30)	412 (0.28)	423 (0.28)	403 (0.25)	452 (0.27)	318 (0.19)	214 (0.13)
50-59	3,128 (2.64)	3,523 (2.80)	3,370 (2.67)	3,097 (2.54)	3,193 (2.44)	3,134 (2.34)	3,002 (2.15)	2,983 (2.03)	2,809 (1.94)	2,398 (1.67)
60-69	7,852 (9.86)	8,510 (10.08)	8,014 (9.45)	7,202 (8.81)	7,235 (8.22)	6,988 (7.76)	6,989 (7.46)	6,814 (6.90)	6,345 (6.51)	6,213 (6.43)
70-79	8,496 (16.92)	9,476 (17.81)	8,690 (16.25)	7,812 (15.15)	7,529 (13.57)	7,297 (12.86)	7,404 (12.53)	7,322 (11.76)	6,841 (11.14)	6,693 (10.98)
80-89	2,995 (13.73)	3,249 (14.06)	2,881 (12.40)	2,536 (11.32)	2,671 (11.08)	2,569 (10.42)	2,668 (10.39)	2,713 (10.03)	2,670 (10.01)	2,746 (10.37)
90+	62 (2.37)	76 (2.75)	67 (2.41)	52 (1.94)	56 (1.94)	68 (2.30)	69 (2.24)	77 (2.38)	76 (2.38)	97 (3.06)

Rates (presented in parentheses) are age-standardized to the 2006 Australian population at risk in each SES decile.

*SES decile 1 is the most disadvantaged.

**Figure 1:**
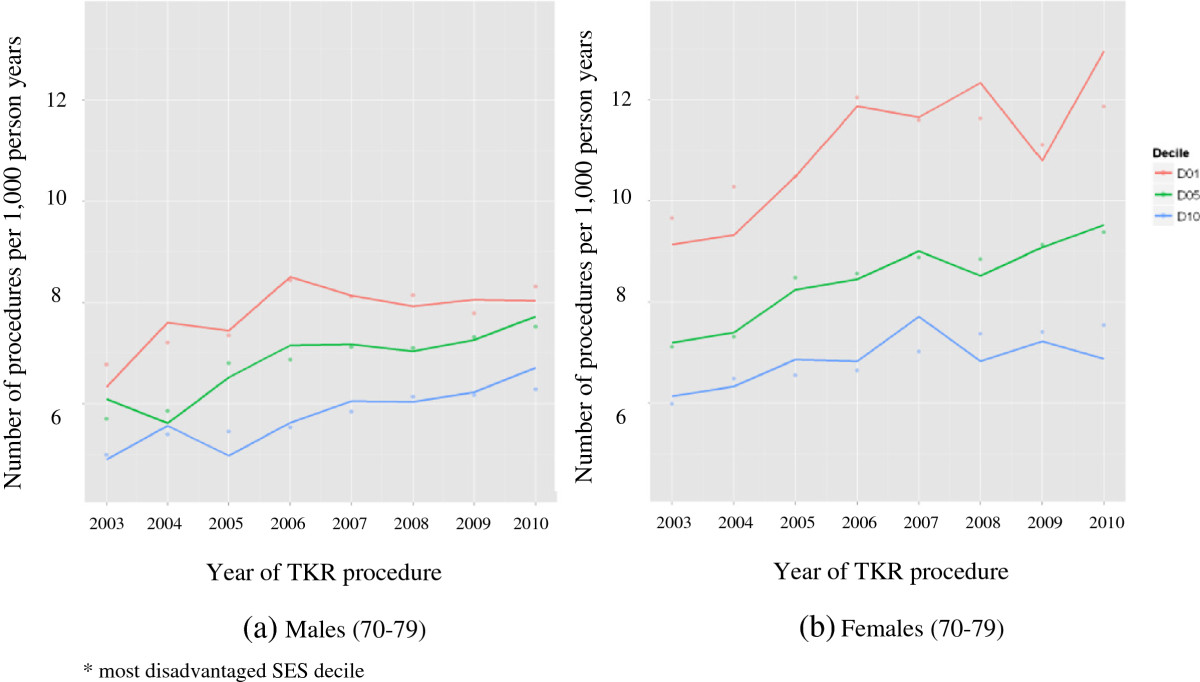
Observed (lines) and estimated (dots) rates per 1,000 person years of primary total knee replacement (TKR) across years 2003–10 (in the 70–79 year age group) by deciles of SES (only the first*, fifth and tenth SES decile presented) in (a) males and (b) females.

**Figure 2:**
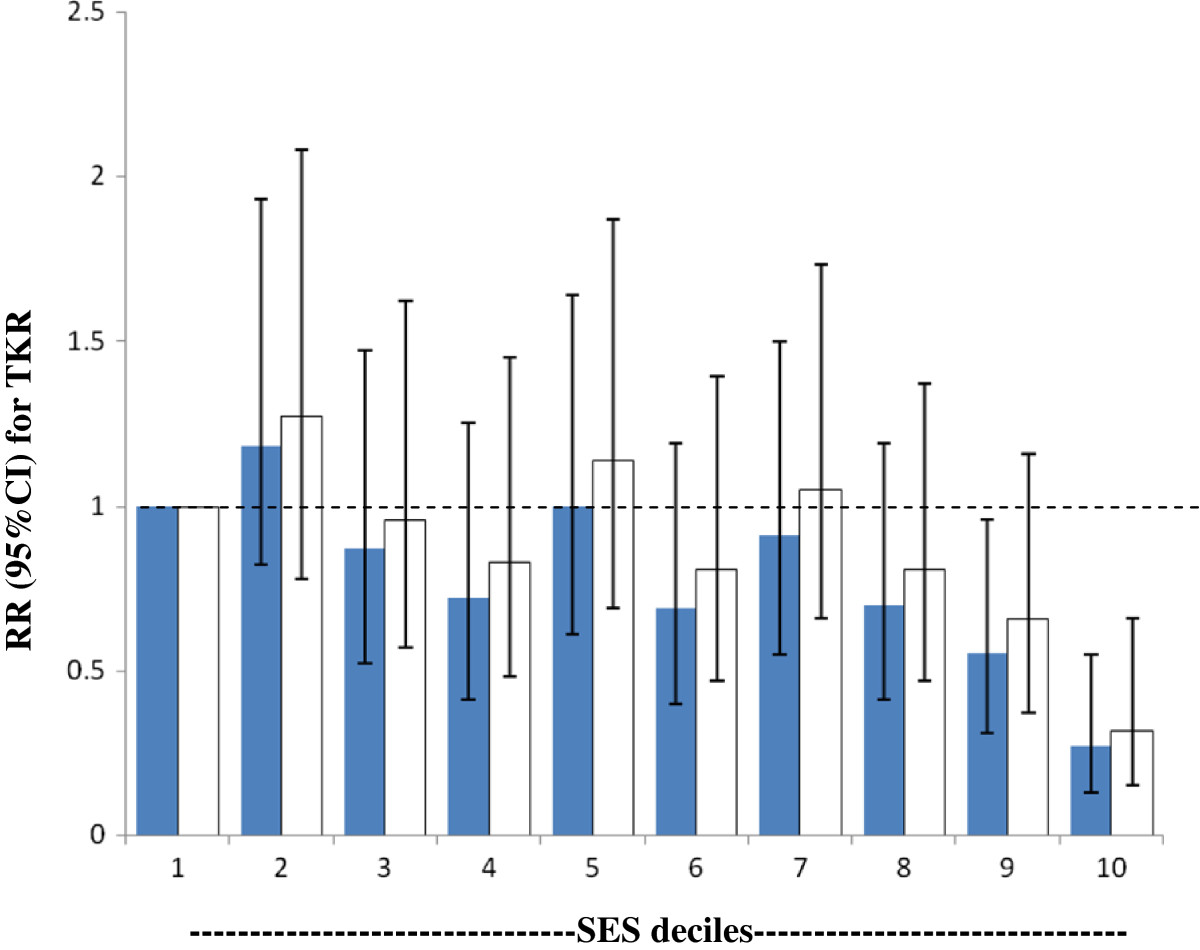
**Relative rates (RR) for primary total knee joint replacement (TKR) for males and females 2003–10 in SES deciles 2–10 compared to SES decile 1 (most disadvantaged), for any age or year.**

Given that the greatest surgery utilisation was observed in men and women aged 70–79 years, and in order to examine whether TKR varied according to year of surgery, Table 2 presents the estimated rates of TKR per 1,000 person years for procedures performed 2003–10 for males and females in that age group in the lowest and highest SES decile. For this age group, estimated rates of TKR increased from 2003 to 2010 for SES decile 10 (most advantaged) and decile 1 (most disadvantaged); furthermore, females had a greater rate of TKR compared with males. The 60–69 and 80–89 year age groups showed similar patterns.Figure 1 presents the observed rates of TKR over time by SES deciles for males and females in the 70–79 year age group. A greater increase in TKR utilisation over the time period was observed in females than in males. The negative relationship between TKR rates and SES persisted over the study period.Figure 2 presents the relative risk (RR) and 95% confidence intervals (95%CI) for primary TKR between SES deciles for females and males, at any age or year. Poisson regression showed RR >1 in SES deciles 2, 5 and 7 in primary TKR; with the greatest rate of TKR observed in the most disadvantaged SES decile and the lowest rate observed for the most advantaged decile. Although lower rates of primary TKR were seen for most deciles compared to decile 1, the 95%CI all indicated non-significance with the exception of the most advantaged (decile 10) and for females in decile 9.

**Table 2:** **Estimated rates (95%CI) of primary TKR per 1,000 person years 2003–10 for the lowest and highest decile of socioeconomic status (SES) for males and females aged 70–79 years**

Year	Females		Males	
	SES decile 1*	SES decile 10	SES decile 1*	SES decile 10
2003	9.66 (9.19, 10.15)	5.99 (5.66, 6.33)	6.77 (6.43, 7.13)	4.99 (4.71, 5.29)
2004	10.28 (9.80, 10.78)	6.48 (6.14, 6.84)	7.20 (6.86, 7.57)	5.40 (5.11, 5.70)
2005	10.48 (10.01, 10.98)	6.54 (6.21, 6.90)	7.35 (7.00, 7.71)	5.45 (5.17, 5.76)
2006	12.04 (11.52, 12.58)	6.64 (6.31, 6.99)	8.44 (8.06, 8.84)	5.53 (5.25, 5.83)
2007	11.59 (11.09, 12.11)	7.02 (6.68, 7.37)	8.12 (7.76, 8.50)	5.85 (5.56, 6.15)
2008	11.62 (11.14, 12.13)	7.37 (7.03, 7.72)	8.15 (7.79, 8.52)	6.14 (5.85, 6.44)
2009	11.11 (10.64, 11.59)	7.41 (7.08, 7.76)	7.79 (7.45, 8.14)	6.17 (5.89, 6.47)
2010	11.86 (11.39, 12.36)	7.54 (7.21, 7.89)	8.32 (7.97, 8.68)	6.28 (6.00, 6.58)

*Most disadvantaged SES decile.

## Discussion

We report an overall decrease in TKR utilisation with increased SES. Those in the most advantaged group were less likely to undergo a TKR than the most disadvantaged group. Estimated rates of TKR increased more sharply in females than in males, and the observed rates of primary TKR also showed a steeper increase over time in females than males; patterns that plausibly reflect the greater prevalence of OA in women compared with men.

Our study examined primary TKR utilization, a factor that is linked to health-seeking behaviour. Given that those at greatest need of TKR due to OA are in the lower SES groups, we would expect to see increased utilisation of TKR in these population groups. Indeed, the difference in the uptake of TKR between SES deciles is similar to that observed in a smaller Australian study of 27,872 TKR [23], and similar to studies from the UK [7, 13], and indicative of the well-documented social gradient of health. That we observed greater primary TKR uptake in those individuals who would be expected to have greater need, may suggest that socially disadvantaged patients do not appear to be disproportionately disadvantaged by the Australian healthcare system compared with those who are more advantaged. However, care should be taken in suggesting that all individuals, regardless of SES, are supported equitably by the mix of public and private healthcare coverage in the Australian health system with regards to TKR. The cost of TKR surgery in Australia, given that it is considered an elective procedure, has been shown to influence patient preferences, whereby having private health insurance, rather than income alone, predicted uptake [32]. Furthermore, it is plausible that socially advantaged individuals may have greater financial resources to access other modes of management for OA than TKR; modes that may include flexibility in work-related activities, physiotherapy, analgesics, and early retirement. Conversely, socially disadvantaged individuals are reported to have lower health literacy compared to those with greater educational attainment and/or income [33, 34], which may in turn influence their preferences and willingness to utilise surgery [25, 35]. Adding complexity to the association between TKR and SES is that whilst health literacy declines with age [36–38], co-morbidities increase with age. Medical professionals and patients may have expectations of poorer outcomes [39], especially where co-morbidities exist [40]; notably it is those of lower SES that are more likely to have comorbid conditions and less healthy lifestyle behaviours that their socially advantaged counterparts [41]. Surgery uptake may also be associated with the severity of knee OA, and the willingness of the patient to consider surgery [39]; factors that may be especially pertinent for those of lower SES [25, 42].

Clearly linked to health-seeking behaviour and health literacy are life circumstances that enable individuals the opportunity to choose whether to undergo surgery. Different social groups have disparate social and economic imperatives that may impact on choice to undergo a primary TKR. For instance, socially disadvantaged individuals in Australia may be limited by vacancies on a public health system waiting list, whilst in contrast a more advantaged individual who has private health coverage may be limited by personal schedule alone and would have greater ability to choose an appropriate time to undergo surgery. Australia has a unique healthcare system, however data regarding private versus public sector were not included in these analyses, and thus we are unable to comment further.

Differences in healthcare systems between countries, or an over utilization of TKR in some groups, may explain why others report alternate findings to our current study, whereby lower TKR uptake may be seen for disadvantaged individuals compared to their more advantaged counterparts [43]. It is also plausible that socially advantaged individuals may cope with end-stage disease and related pain for a longer time period than more socially disadvantaged individuals. For instance, it is suggested that individuals of higher SES are more likely to have better pre-operative function [8, 44] (potentially also related to lower rates of obesity observed in higher SES groups in Australia [41, 45, 46]), and also increased coping mechanisms than those of lower SES. Thus, while our data appear to reflect greater TKR uptake among those expected to have greater need, we are unable to determine whether this reflects actual variations in need or in clinical practice and recommendations for surgery [47], or whether the time differs across SES between an identified need for surgery and actual surgery utilisation. Understanding the association between SES and TKR utilisation has clear policy implications for appropriate allocation of health services and resources across the SES spectrum, for instance improving access to primary healthcare and/or multidisciplinary healthcare expertise, an/or targeting those at greatest need.

It is important to acknowledge that, beyond age and sex, obesity is one of major risk factors for knee OA [48–52]. Furthermore, the association between obesity and SES has been well documented in many countries [41, 45, 53, 54]. Taken in context, we may speculate that obesity may be associated with help-seeking behaviours related to a TKR, however, it is not known whether TKR surgery is more likely taken up by obese compared with non-obese individuals in this Australian population. Obesity may be an important factor in selection for surgery as some orthopaedic surgeons may refuse to perform TKR unless weight loss has occurred; surgical outcomes are generally poorer in obese patients with greater perioperative morbidity and mortality [55, 56]. Studies have shown that the most positive outcomes post-surgery are observed for patients with greater pre-operative function [57], higher pre-operative expectations [58] where disease pre-surgery is less severe [57]; factors more likely seen in non-obese rather than obese patients, and therefore higher SES groups compared to lower SES groups. However, a review suggested that obesity did not adversely affect the longevity of prosthesis, and therefore was not a predictor of revision rates [59].

Our study has various strengths. Our analyses included all TKR performed for OA, Australia-wide over an eight-year period, from a comprehensive national registry that has been validated against health department unit record data using a sequential multi-level matching process. Coupled with the retrieval of unreported procedures, the Registry is the most complete set of data relating to joint replacement surgeries in Australia. However, by using administrative data, we are limited to examining those that underwent TKR, but not those that needed TKR. This study also has some limitations. We were unable to account for whether primary TKR were performed in the public or private sector. However, the aim of this study was to examine utilization of primary TKR surgery rather than accessibility to surgery, which may be increased for those who have private health coverage compared to those reliant on the public health care system. The IRSAD is an aggregate of various individual parameters of SES and is formed into an area-based measure of SES from data collected as part of the Australian Census. The use of an aggregate SES index assumes that relatively disadvantaged individuals do not reside in areas of upper SES, and vice versa for relatively advantaged individuals in areas of lower SES. For these analyses, we pooled individuals into deciles based on SEIFA values from two different census periods. We acknowledge that the IRSAD is not strictly comparable across different time periods given that different parameters are used to form aggregate measures at different census periods, however, a comparison between 2001 and 2006 census data shows that the mean change across all comparable CCDs was constrained within expected limits for every variable [27]. We made the assumption that population growth within SES deciles occurred at the same rate over the study period; however, it is possible that this may have introduced some error into the calculations. Nevertheless, we speculate that this may result in only a small under- or over-estimation of associations. We were unable to examine any ethnic or cultural differences in TKR, as these data are not collected by the AOA NJRR; however, we recognize that ethnic differences may exist, as reported by previous studies [60–62].

## Conclusions

In conclusion, we observed differences in the uptake of TKR that reflected the well-documented social gradient of health. However, further work is required to elucidate whether social disparities exist in the time between identifying the need for surgical intervention and actual surgery uptake. It is imperative that factors entangled with the association between SES and TKR utilisation are understood, as these have clear implications for appropriate allocation of health resources across the SES spectrum.

## Authors’ original submitted files for images

Below are the links to the authors’ original submitted files for images. Authors’ original file for figure 1 Authors’ original file for figure 2

## Abbreviations

ABS: Australian Bureau of Statistics
AOA NJRR: Australian Orthopaedic Association National Joint Replacement Registry
CCD: Census Collection District
IRSAD: Index for Relative Socioeconomic Advantage and Disadvantage
OA: Osteoarthritis
PAR: Population At Risk
RR: Relative Risk
TKR: Total Knee Replacement
SEIFA: SocioEconomic Index For Areas
SES: Socioeconomic Status.
