# Supplementary figures and images for: Associations between socioeconomic status and primary total knee joint replacements performed for osteoarthritis across Australia 2003–10: data from the Australian Orthopaedic Association National Joint Replacement Registry
Source: BMC Musculoskelet Disord. 2014 Oct 28;15:356. doi: 10.1186/1471-2474-15-356 (PMC4223827; doi:10.1186/1471-2474-15-356)

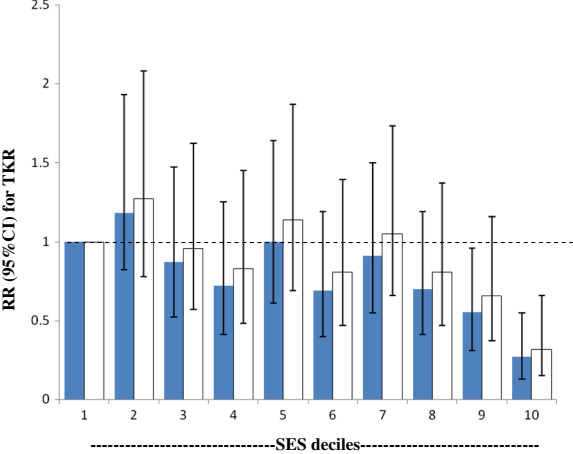

Supplement: Supplementary file 2 — Authors’ original file for figure 2 [file 12891_2014_2293_MOESM2_ESM.pdf]
